# Development of a nomogram prediction model for postoperative intra-abdominal hypertension in patients with intra-abdominal infection sepsis based on analgesic drug selection

**DOI:** 10.3389/fsurg.2026.1899876

**Published:** 2026-09-11

**Authors:** Wenjing Zhao, Guoqiang Chen, Yan Xu, Diyin Yang, Qiaozhen Zhang

**Affiliations:** 1 Department of Critical Care Medicine, The Affiliated Hospital of Xuzhou Medical University, Xuzhou, China; 2 Department of Anesthesiology, The Affiliated Hospital of Xuzhou Medical University, Xuzhou, China

**Keywords:** intra-abdominal hypertension, nomogram, oxycodone, postoperative complications, sepsis

## Abstract

**Background:**

Postoperative intra-abdominal hypertension (IAH) is a common complication in patients with sepsis secondary to intra-abdominal infection and is associated with organ dysfunction and prolonged intensive care unit (ICU) stay. This study aimed to identify risk factors for postoperative IAH and develop a nomogram incorporating analgesic strategy and clinical variables for early risk stratification.

**Methods:**

This retrospective cohort study included adult patients who underwent surgery and received postoperative opioid analgesia in the ICU between June 2024 and October 2025. The primary outcome was postoperative IAH, defined as an intra-abdominal pressure >12 mmHg occurring within 48 h after initiation of postoperative opioid analgesia in the ICU. Multivariable logistic regression and LASSO regression were used to identify independent predictors, and a nomogram was subsequently developed. Model performance was evaluated using the area under the receiver operating characteristic curve (AUC), calibration plots, and decision curve analysis.

**Results:**

Among 116 patients, 49 developed IAH. Compared with patients without IAH, those with IAH were older (*P* = 0.027), had higher body mass index (BMI) (*P* = 0.006), lower albumin (*P* = 0.018), higher lactate (*P* < 0.001), and had a lower rate of oxycodone administration (*P* = 0.033). Multivariate analysis identified age (OR = 1.07, *P* = 0.011), BMI (OR = 1.21, *P* = 0.019), and lactate (OR = 1.22, *P* = 0.035) as risk factors, while oxycodone use (OR = 0.39, *P* = 0.033) and albumin (OR = 0.92, *P* = 0.011) were protective. The nomogram showed good discrimination (AUC = 0.783) and calibration.

**Conclusions:**

Advanced age, elevated BMI, and higher lactate levels were independently associated with increased odds of postoperative IAH in sepsis patients, whereas postoperative oxycodone use was independently associated with a lower odds of postoperative IAH. The nomogram may facilitate early identification of high-risk patients and guide perioperative management.

## Background

Secondary peritonitis caused by gastrointestinal perforation, intestinal obstruction, or ischemic injury remains a major cause of sepsis and critical illness worldwide. In these patients, disruption of the intestinal barrier permits bacteria, endotoxins, and inflammatory mediators to enter the systemic circulation, triggering a dysregulated host response that may lead to multiple organ dysfunction if timely intervention is not provided (1, 2). One important but often underrecognized complication in this setting is intra-abdominal hypertension (IAH), which results from intestinal edema, paralytic ileus, capillary leak, and aggressive fluid resuscitation (3). Previous studies have reported that IAH occurs in up to 40%–60% of critically ill patients with sepsis and is associated with prolonged mechanical ventilation, acute kidney injury, and increased mortality (4, 5). Therefore, early identification of patients at high risk for postoperative IAH is clinically important for optimizing perioperative management and improving patient outcomes.

The pathophysiological relationship between sepsis and IAH is complex and multifactorial. Systemic inflammation leads to mesenteric hypoperfusion, endothelial dysfunction, and increased vascular permeability, resulting in bowel wall edema and intra-abdominal fluid accumulation (6, 7). Elevated intra-abdominal pressure (IAP) further compromises venous return, reduces renal perfusion pressure, and restricts diaphragmatic excursion, thereby exacerbating respiratory and renal dysfunction (8). Postoperative pain and agitation may further aggravate this vicious cycle by increasing thoracoabdominal muscle tension and abdominal wall rigidity. Opioid analgesics are routinely administered for postoperative pain control in these patients; however, conventional μ-opioid receptor agonists are known to inhibit gastrointestinal motility, promote postoperative ileus, and worsen abdominal distension, potentially contributing to elevated IAP (9, 10). In contrast, oxycodone has a distinct pharmacological profile characterized by mixed μ- and κ-opioid receptor activity and relatively lower μ-receptor affinity, which may provide advantages in visceral pain control while causing less impairment of gastrointestinal function (11).

Several clinical studies have investigated risk factors and clinical outcomes associated with IAH in critically ill or surgical populations, identifying advanced age, obesity, hypoalbuminemia, hyperlactatemia, and positive fluid balance as important contributing factors (12, 13). Meanwhile, emerging evidence suggests that oxycodone provides effective visceral analgesia and may attenuate inflammatory responses compared with other opioids, particularly following abdominal and gastrointestinal surgery (11). However, previous studies have primarily focused on analgesic efficacy, postoperative pain intensity, or inflammatory markers rather than postoperative IAH as a clinically relevant outcome (14, 15). Furthermore, most investigations have evaluated individual risk factors in isolation without incorporating analgesic strategy into an integrated predictive framework. To our knowledge, no validated prediction model has incorporated postoperative analgesic selection to estimate the risk of postoperative IAH in patients with sepsis secondary to intra-abdominal infection.

Given these limitations, there is a need for a clinically applicable tool that integrates analgesic strategy with readily available perioperative variables to facilitate early risk stratification. Nomograms have gained increasing attention as intuitive and individualized prediction tools that enable clinicians to quantify patient-specific risk and support bedside decision-making (16). Therefore, the objective of this study was to develop a nomogram prediction model incorporating postoperative analgesic selection and key clinical variables to predict postoperative IAH in patients with intra-abdominal infection–related sepsis. Early identification of patients at high risk for postoperative IAH may facilitate individualized perioperative management and timely implementation of targeted interventions.

## Methods

### Subjects

This retrospective cohort study was approved by the Medical Ethics Committee of the Affiliated Hospital of Xuzhou Medical University (XYFY2025-KL525-01) and registered with the Chinese Clinical Trial Registry (ChiCTR2500112016). Patients who underwent surgery for gastrointestinal perforation, intestinal obstruction, and other related conditions between June 2024 and October 2025 were consecutively enrolled.

All patients met the Sepsis-3 diagnostic criteria (17) and were admitted to the intensive care unit (ICU). Sepsis-3 was defined as suspected or documented infection accompanied by an acute increase in the Sequential Organ Failure Assessment (SOFA) score of ≥2 points attributable to infection. The SOFA score was calculated using the worst clinical and laboratory parameters recorded within the first 24 h surrounding ICU admission. For patients without known pre-existing organ dysfunction, the baseline SOFA score was considered to be 0; for patients with documented chronic organ dysfunction, baseline values were determined according to available pre-admission clinical records.

Inclusion criteria were as follows: patients of either sex; age ≥18 years; postoperative incisional pain, agitation, delirium, or intolerance of various tubes requiring analgesic and sedative therapy; and American Society of Anesthesiologists (ASA) physical status I–III. Patients with ASA physical status IV–V were excluded because these patients often present with severe preoperative physiological instability, multiple organ dysfunction, or immediately life-threatening conditions, which may introduce substantial heterogeneity in perioperative management and analgesic selection.

Exclusion criteria were: (1) a history of psychiatric medication use or alcohol dependence; (2) severe cardiovascular, cerebrovascular, renal, or hepatic disease before admission; (3) use of other sedative or analgesic interventions within one week before enrollment; (4) schizophrenia, epilepsy, Parkinson’s disease, or myasthenia gravis; and (5) opioid dependence, opioid allergy, or contraindications to the study medications. Given the retrospective study design and the use of anonymized data, the requirement for written informed consent was waived by the Ethics Committee.

The primary surgical diagnoses included gastrointestinal perforation, intestinal obstruction, intestinal ischemia or necrosis, anastomotic leakage, and other intra-abdominal conditions requiring emergency surgery. The principal surgical procedures included perforation repair, bowel resection with primary anastomosis, bowel resection with stoma formation, exploratory laparotomy or laparoscopy, abdominal drainage, and other procedures performed according to the underlying pathology and intraoperative findings.

### Postoperative analgesia

After ICU admission, all patients received continuous intravenous opioid analgesia for postoperative pain management. The analgesic strategy was determined according to clinical requirements and CPOT scores. Patients were categorized according to the primary opioid regimen: oxycodone-based analgesia or remifentanil-based analgesia. The oxycodone regimen consisted of oxycodone hydrochloride (1.5 mg/kg diluted to 50 mL with normal saline) administered by continuous intravenous infusion at 0.03–0.06 mg/kg/h. The remifentanil regimen consisted of remifentanil (50 μg/kg diluted to 50 mL with normal saline) administered by continuous intravenous infusion at 1.0–2.0 μg/kg/h. Patients classified as not receiving oxycodone received remifentanil-based analgesia as their primary postoperative opioid regimen. Infusion was continued during the postoperative ICU period according to analgesic requirements and was gradually discontinued after clinical reassessment when patients were conscious, had recovered respiratory function, and had stable hemodynamics.

Rescue fentanyl administration was recorded as a binary variable (yes/no), defined according to whether at least one rescue dose was administered during the first 48 h after initiation of postoperative analgesia. Because cumulative fentanyl dose and the exact number of rescue administrations were not consistently available in the retrospective records, these variables were not included in the analysis.

### Data acquisition

Preoperative data collected within 24 h before surgery were extracted from the electronic medical record system. Demographic characteristics included age, sex, height, weight, and body mass index (BMI). Clinical variables comprised ASA classification, smoking history, alcohol consumption history, and comorbidities, including hypertension, diabetes mellitus, chronic obstructive pulmonary disease (COPD), and a history of cerebral infarction.

Baseline laboratory parameters included hemoglobin (HGB), albumin (ALB), total bilirubin (T-Bil), alanine aminotransferase (ALT), aspartate aminotransferase (AST), serum creatinine (Cr), N-terminal pro-B-type natriuretic peptide (NT-proBNP), white blood cell count (WBC), neutrophil count (NEUT), lymphocyte count (LYMPH), platelet count (PLT), C-reactive protein (CRP), and serum lactate. The oxygenation index was also recorded.

Laboratory variables used for prediction modeling, including serum albumin, serum lactate, and other biochemical and hematological parameters, were defined as the most recent measurements obtained after ICU admission and immediately before initiation of postoperative opioid analgesia. Laboratory measurements obtained after initiation of opioid analgesia, including those obtained 48 h later, were not used as predictors. Postoperative variables were collected within 48 h after initiation of opioid analgesia in the ICU. IAP, CRP, serum creatinine, and serum lactate were measured before opioid administration and again 48 h after opioid initiation. Additional perioperative management data included operative duration, surgical approach, albumin supplementation volume, 48-h net fluid balance, vasoactive drug use (converted to norepinephrine equivalent dose), and frequency of rescue analgesia. Rescue fentanyl administration was recorded as a binary variable (yes/no) according to whether at least one rescue dose was administered during the first 48 h after initiation of postoperative analgesia. The incidence of nausea and vomiting was recorded after patients regained full consciousness, defined as a Richmond Agitation-Sedation Scale (RASS) score of 0, characterized by spontaneous eye opening and the ability to follow simple commands such as hand squeezing or arm lifting.

### Outcomes and assessments

The primary outcome was postoperative IAH occurring within 48 h after initiation of postoperative opioid analgesia in the ICU. IAP was measured before opioid administration and monitored during the subsequent 48-h observation period and via bladder pressure monitoring as follows. With the patient in the supine position, a specialized urinary catheter was inserted into the bladder through the urethra under sterile conditions. After the bladder was emptied, the three-way stopcock was adjusted to instill up to 25 mL of sterile saline into the bladder through the catheter. The catheter was then clamped, and the three-way stopcock was readjusted to connect the catheter to atmospheric pressure. Using the midaxillary line at the level of the iliac crest as the zero reference point, the height of the water column was measured to determine the IAP. An IAP >12 mmHg was defined as IAH (18). Other clinical outcomes included acute kidney injury (AKI), duration of mechanical ventilation, length of postoperative ICU stay, postoperative hospital stay, and in-hospital mortality.

All patients underwent IAP measurement upon admission to the ICU. To ensure a clear temporal relationship between opioid exposure and outcome, only patients in whom opioid analgesia was initiated before the occurrence of IAH were included in the final analysis.

### Sample size calculation

The sample size for this retrospective study was determined by the number of eligible patients available during the study period rather than by an *a priori* sample size calculation. A total of 49 patients developed postoperative IAH. Five predictors were retained in the final multivariable logistic regression model, corresponding to an events-per-variable (EPV) of approximately 9.8. Although this value is close to the conventional benchmark of 10 events per variable, the initial consideration of 26 candidate predictors relative to 49 outcome events indicates a potential risk of model overfitting. Therefore, the model was internally validated using 1,000 bootstrap resampling iterations, and LASSO regression with 10-fold cross-validation was performed as a supplementary analysis to assess the consistency of predictor selection. The limited sample size and absence of external validation were considered important limitations of the model.

### Statistical analysis

Statistical analysis was conducted using IBM SPSS 26.0, R software, and MedCalc software. Missing data patterns were assessed for all variables included in the analysis. The primary outcome (postoperative IAH) had no missing values and was not imputed. Variables with a missing rate >20% were excluded from the analysis, whereas variables with a missing rate <20% were imputed. For predictor variables with missing observations, the missingness mechanism was assumed to be missing at random (MAR), and multiple imputation by chained equations (MICE) was performed. Twenty imputed datasets were generated, incorporating all candidate predictors and the outcome indicator into the imputation model. Estimates from the imputed datasets were pooled using Rubin’s rules. Variables without missing values were analyzed using the original observed data (Supplementary Table S1). Categorical data are presented as counts and percentages, and differences between groups were compared using the chi-square test. Normality of continuous variables was assessed using the Shapiro–Wilk test. Normally distributed data are expressed as the mean ± standard deviation (SD), and the independent-samples *t*-test was used to evaluate between-group differences. Non-normally distributed data are presented as the median (interquartile range) and were analyzed using the Mann–Whitney *U* test or Kruskal–Wallis test, as appropriate. Candidate predictors were prespecified based on clinical relevance, previously reported associations with intra-abdominal hypertension or critical illness, and availability in routine perioperative practice. Univariable logistic regression analyses were initially performed to examine the associations between candidate predictors and postoperative IAH. Univariable analyses were used as an initial screening step to identify variables for consideration in the multivariable model. Given the limited number of outcome events, LASSO regression with 10-fold cross-validation was additionally performed as a sensitivity analysis. Results are reported as odds ratios (ORs) with 95% confidence intervals (CIs). Before variable selection, collinearity was assessed by calculating the variance inflation factor (VIF). A VIF >10 indicated severe multicollinearity, which was addressed by merging overlapping variables or excluding highly collinear variables.

The predictive performance of the model was evaluated using the area under the receiver operating characteristic (ROC) curve (AUC), and comparisons of AUCs were performed using the DeLong test. R software was used to construct the nomogram. Model stability was evaluated using 1,000 bootstrap resampling iterations. ROC analysis was used to evaluate model discrimination, whereas calibration was assessed using the calibration curve and the Hosmer-Lemeshow test. Decision curve analysis (DCA) was performed to evaluate the clinical utility of the model by estimating the net benefit across threshold probabilities ranging from 0.1 to 0.9. As a supplementary sensitivity analysis, least absolute shrinkage and selection operator (LASSO) regression was performed to screen 26 candidate predictors. The purpose was to assess the stability of variable selection under penalized regression and to verify the robustness of the predictors identified by the conventional multivariable logistic regression model. A two-sided *P* < 0.05 was considered statistically significant.

## Results

**Figure 1:**
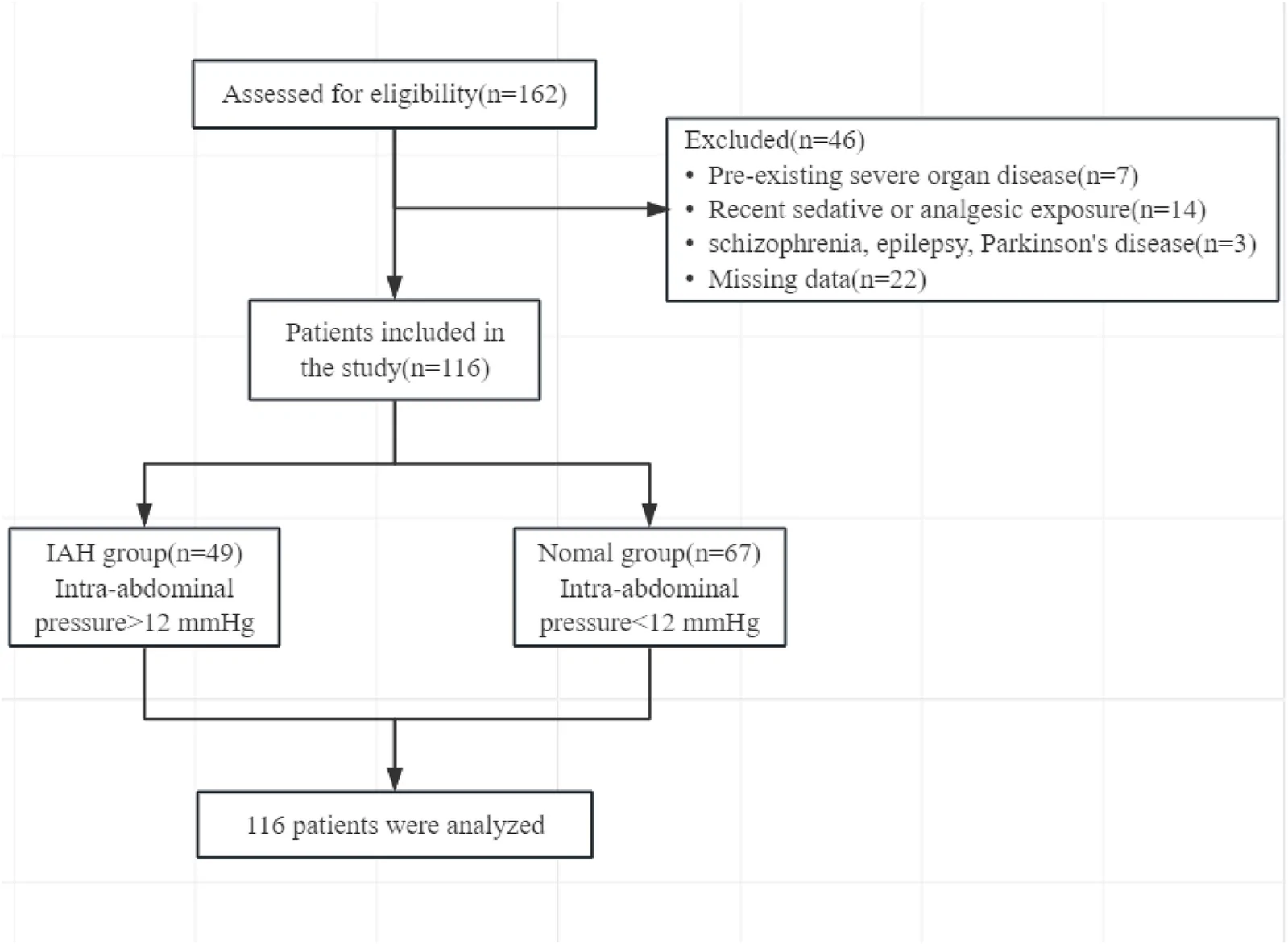
Study flowchart.

A total of 116 patients were included, comprising 49 in the IAH group and 67 in the normal group (Figure 1). Patients who developed IAH were significantly older than those without IAH (70.4 ± 8.7 vs. 66.7 ± 9.6 years, *P* = 0.027) and had a higher BMI (23.6 ± 3.3 vs. 22.0 ± 3.1 kg/m^2^, *P* = 0.006). Serum albumin levels were significantly lower in the IAH group (27.1 vs. 30.1 g/L, *P* = 0.018), while total bilirubin levels were lower (8.8 vs. 12.1 μmol/L, *P* = 0.027). Serum lactate was markedly elevated in the IAH group (3.5 vs. 1.8 mmol/L, *P* < 0.001). The proportion of patients receiving oxycodone was significantly lower in the IAH group than in the non-IAH group (32.7% vs. 59.7%, *P* = 0.033). No significant between-group differences were observed in sex distribution, smoking or alcohol use, comorbidities (hypertension, diabetes, COPD, and cerebral infarction), NT-proBNP, hemoglobin, liver enzymes, creatinine, inflammatory markers (WBC, neutrophils, lymphocytes, and CRP), platelet count, oxygenation index, albumin supplementation, operative duration, surgical approach, rescue fentanyl analgesia, or 48-h net fluid balance (all *P* > 0.05) (Table 1).

**Table 1:** Perioperative characteristics comparison between groups.

Indicator	IAH Group (*n* = 49)	Normal Group (*n* = 67)	*P* value
Age, years	70.4 ± 8.7	66.7 ± 9.6	0.027
Female, *n* (%)	19 (38.8)	27 (39.1)	0.868
BMI, kg/m^2^	23.6 ± 3.3	22.0 ± 3.1	0.006
Smoking, *n* (%)	11 (22.4)	14 (20.9)	0.841
Alcohol use, *n* (%)	5 (12.2)	10 (14.9)	0.454
Hypertension, *n* (%)	6 (12.2)	16 (23.9)	0.114
Diabetes mellitus, *n* (%)	6 (12.2)	9 (13.4)	0.851
COPD, *n* (%)	7 (14.3)	10 (14.9)	0.923
Cerebral infarction, *n* (%)	11 (22.4)	17 (25.4)	0.716
NT-proBNP, pg/mL	2,000 (415.8, 3,608.5)	1,246.5 (395.3, 2,504.8)	0.540
HGB, g/L	107 (91.5, 118)	110 (93, 133)	0.540
ALB, g/L	27.1 (21.2, 30)	30.1 (24.1, 34.2)	0.018
T-Bil, μmol/L	8.8 (6.6, 16.7)	12.1 (8.6, 18.9)	0.027
ALT, U/L	26 (16.5, 39.5)	21 (17, 33)	0.644
AST, U/L	16 (11, 25.8)	15 (12, 26)	0.666
Cr, μmol/L	82 (56, 257.5)	81 (59.5, 149.5)	0.732
WBC, 10^9^/L	8.7 ± 7.7	10.0 ± 8.2	0.380
NEUT, 10^9^/L	7.5 ± 7.3	8.6 ± 8.0	0.418
LYMPH, 10^9^/L	0.7 (0.4, 0.9)	0.8 (0.4, 1.2)	0.292
PLT, 10^9^/L	178.5 ± 87.8	160.2 ± 85.3	0.261
CRP, mg/L	133 (25, 164.1)	31.3 (16.8,164.1)	0.068
Serum lactate, mmol/L	3.5 (2, 5.6)	1.8 (1.3, 3.3)	<0.001
Oxygenation index, mmHg	320.8 ± 122.4	343.9 ± 107.0	0.281
Oxycodone analgesia, *n* (%)	16 (32.7)	40 (59.7)	0.033
Rescue fentanyl analgesia, *n* (%)	12 (24.5)	13 (19.4)	0.265
Open surgery, *n* (%)	13 (26.5)	13 (19.4)	0.363
Operative duration, min	95 (78, 110)	92 (78, 111)	0.969
ALB supplementation, g	80 (45, 145)	75 (0, 120)	0.257
48 h net fluid balance, mL	1,705.8 ± 1,869	1,555.1 ± 1,521.0	0.684

Laboratory variables were based on the most recent measurements obtained before initiation of postoperative opioid analgesia.

IAH, intra-abdominal hypertension; BMI, body mass index; COPD, chronic obstructive pulmonary disease; NT-proBNP, N-terminal pro-B-type natriuretic peptide; HGB, hemoglobin; ALB, albumin; T-Bil, total bilirubin; ALT, alanine aminotransferase; AST, aspartate aminotransferase; Cr, creatinine; WBC, white blood cell; NEUT, neutrophil; LYMPH, lymphocyte; PLT, platelet; CRP, C-reactive protein.

Postoperative outcomes differed between the two groups in several clinically relevant aspects. The incidence of AKI was significantly higher in the IAH group compared with the normal group (51.0% vs. 29.9%, *P* = 0.033). Patients with IAH also required a longer duration of mechanical ventilation (7 vs. 5 days, *P* = 0.013). Although postoperative ICU stay tended to be longer in the IAH group (8 vs. 6 days), the difference did not reach statistical significance (*P* = 0.072). There were no significant differences in postoperative hospital stay (9 vs. 10 days, *P* = 0.955) or in-hospital mortality (28.6% vs. 32.8%, *P* = 0.687) between the two groups (Table 2).

**Table 2:** Postoperative outcomes comparison between groups.

Outcome indicator	IAH group (*n* = 49)	Normal group (*n* = 67)	*P* value
AKI, *n* (%)	25 (51.0)	20 (29.9)	0.033
Mechanical ventilation time, days	7 (3, 10.5)	5 (3, 8)	0.013
Postoperative ICU stay, days	8 (4, 14)	6 (4, 9)	0.072
Postoperative hospital stay, days	9 (5, 14)	10 (5, 13)	0.955
In-hospital mortality, *n* (%)	14 (28.6)	22 (32.8)	0.687

IAH, intra-abdominal hypertension; AKI, acute kidney injury.

Univariate logistic regression analysis showed that age (OR = 1.05, 95% CI: 1.00–1.10, *P* = 0.042), BMI (OR = 1.19, 95% CI: 1.05–1.35, *P* = 0.008), and serum lactate level (OR = 1.32, 95% CI: 1.09–1.59, *P* = 0.003) were associated with a higher odds of postoperative IAH, whereas postoperative oxycodone analgesia (OR = 0.33, 95% CI: 0.15–0.71, *P* = 0.005) and plasma albumin (OR = 0.94, 95% CI: 0.89–0.99, *P* = 0.037) were associated with a lower odds of postoperative IAH. The VIF values for all variables were <10. After including postoperative oxycodone use, age, BMI, plasma albumin, and serum lactate level in the multivariable logistic regression analysis, age (OR = 1.07, 95% CI: 1.01–1.13, *P* = 0.011), BMI (OR = 1.21, 95% CI: 1.00–1.42, *P* = 0.019), and serum lactate level (OR = 1.22, 95% CI: 1.01–1.47, *P* = 0.035) were associated with a higher odds of postoperative IAH in sepsis patients, while postoperative oxycodone analgesia (OR = 0.39, 95% CI: 0.16–0.93, *P* = 0.033) and plasma albumin (OR = 0.92, 95% CI: 0.86–0.98, *P* = 0.011) were associated with a lower odds of postoperative IAH (Table 3).

**Table 3:** Univariate and multivariate logistic regression analysis of postoperative IAH.

Variable	Univariate analysis		Multivariate analysis		
	OR value (95% CI)	*P* value	B	OR value (95% CI)	*P* value
Oxycodone	0.33 (0.15∼0.71)	0.005	−0.950	0.39 (0.16∼0.93)	0.033
Age	1.05 (1.00∼1.10)	0.042	0.067	1.07 (1.01∼1.13)	0.011
BMI	1.19 (1.05∼1.35)	0.008	0.189	1.21 (1.00∼1.42)	0.019
Plasma albumin	0.94 (0.89∼0.99)	0.037	−0.084	0.92 (0.86∼0.98)	0.011
Serum lactate	1.32 (1.09∼1.59)	0.003	0.094	1.22 (1.01∼1.47)	0.035
Constant			−7.103		

Laboratory variables were based on the most recent measurements obtained before initiation of postoperative opioid analgesia. BMI, body mass index.

**Figure 2:**
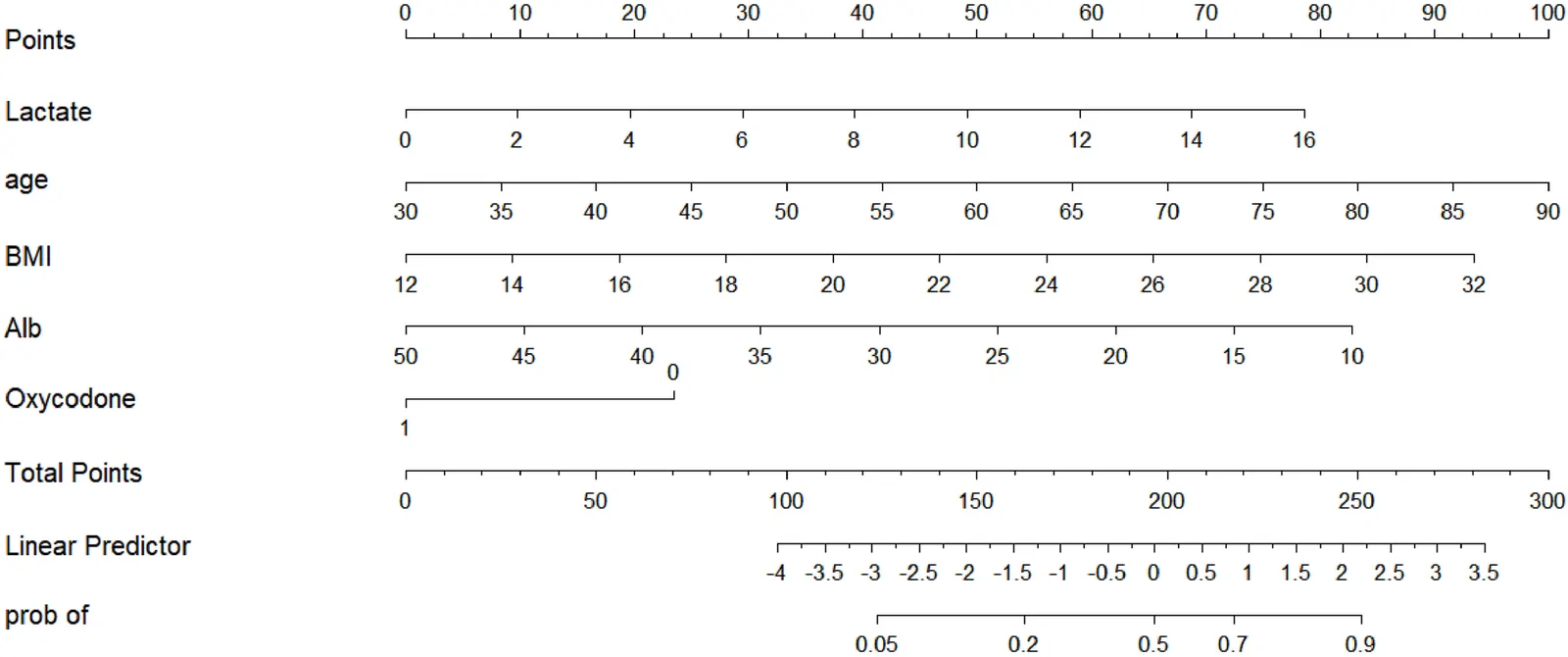
Nomogram prediction model.

**Figure 3:**
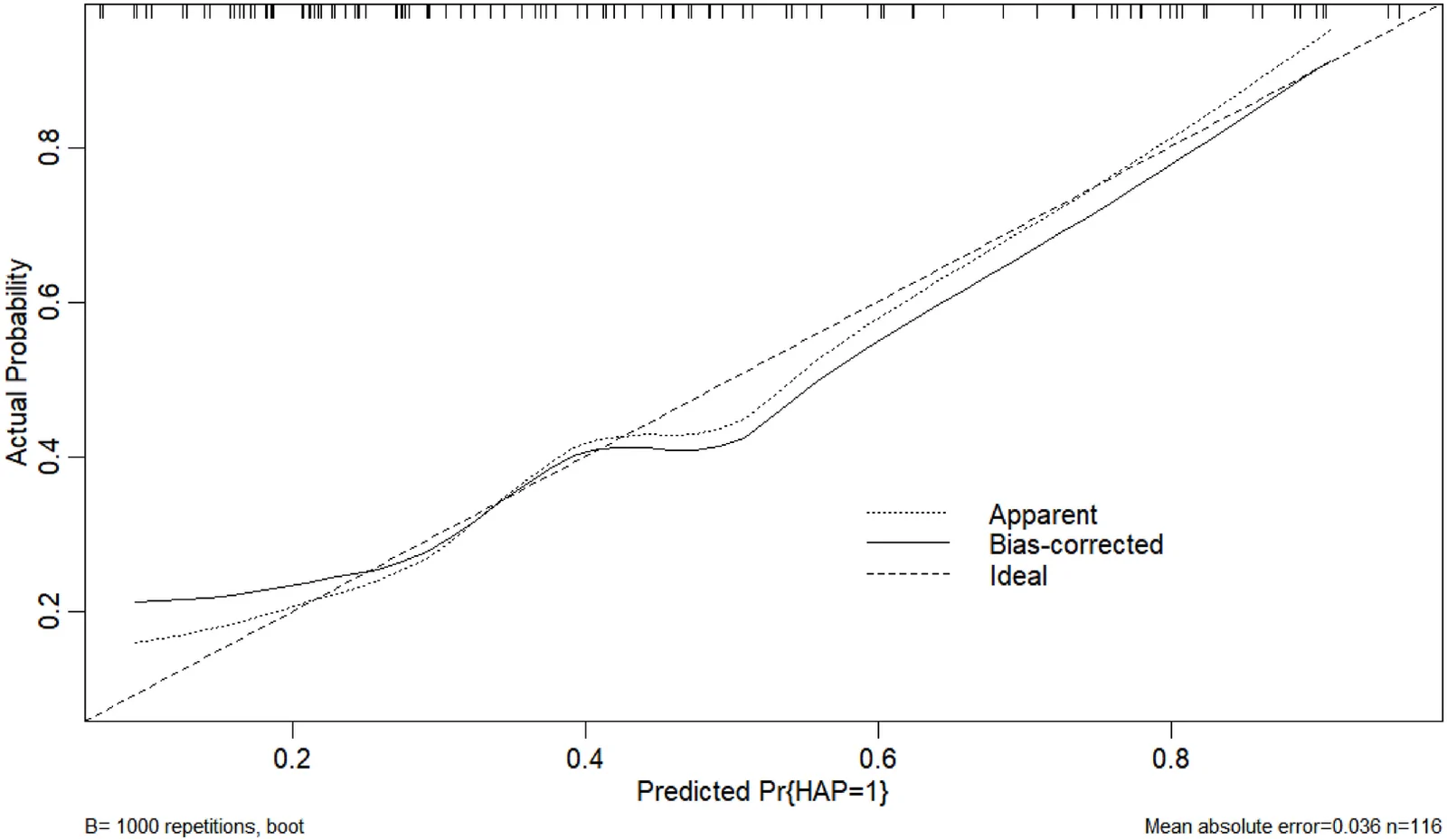
Calibration curve of the prediction model.

**Figure 4:**
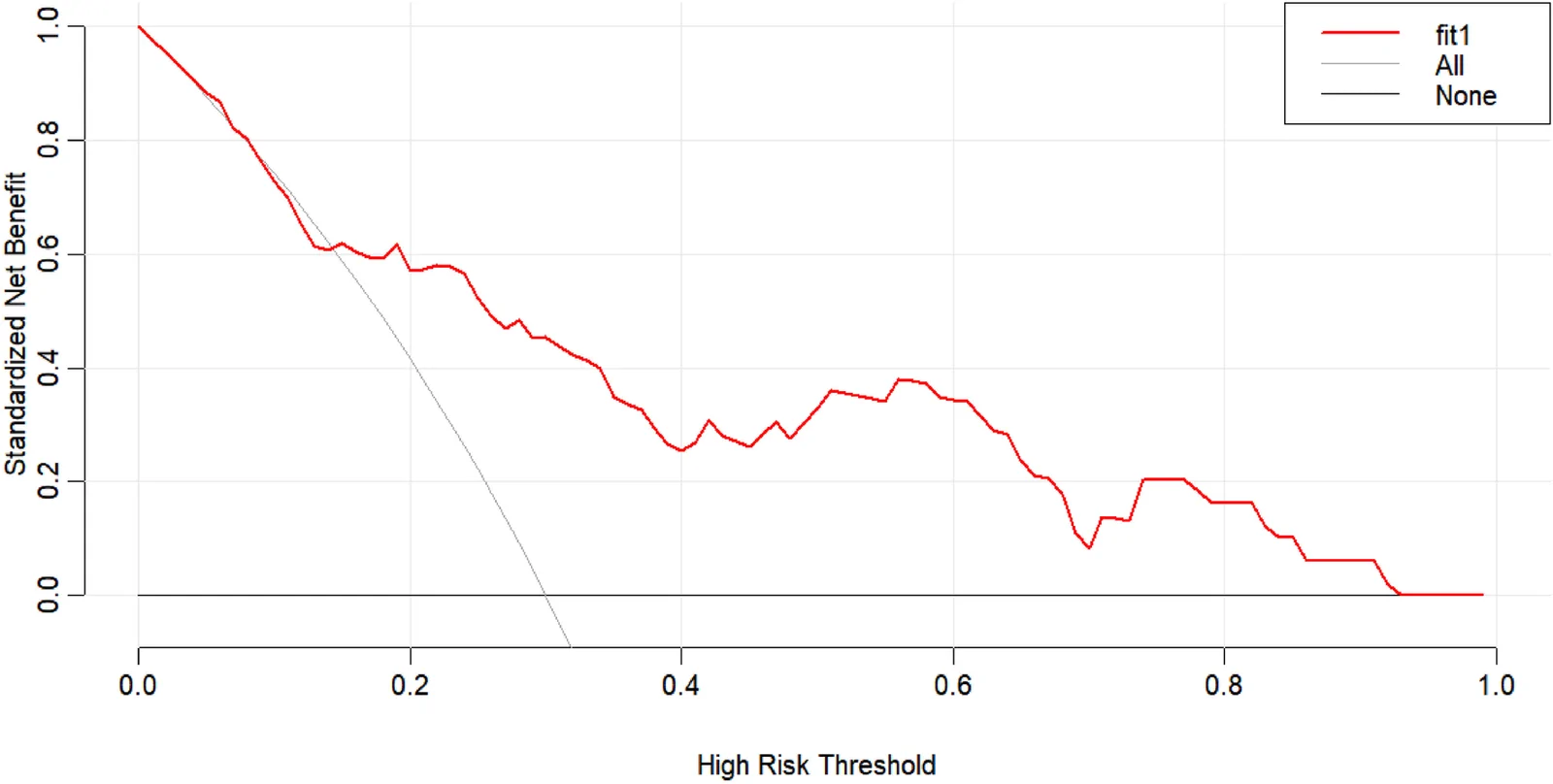
Decision curve of the prediction model.

**Figure 5:**
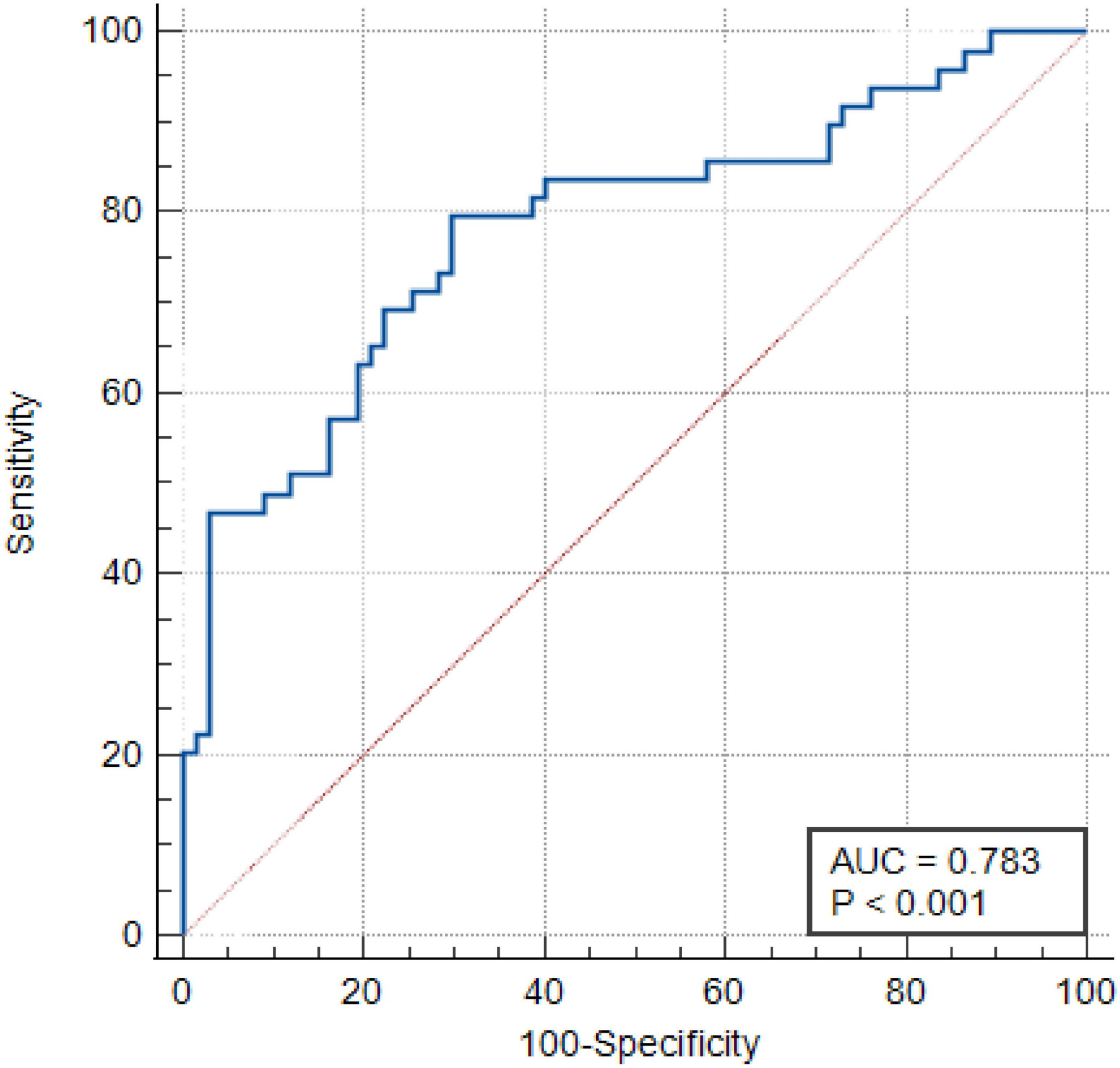
Perioperative risk assessment model for predicting postoperative IAH in septic patients.

To predict postoperative IAH in patients with sepsis, a logistic regression model was constructed based on the independent variables identified in the multivariable analysis, and a corresponding nomogram was subsequently developed (Figure 2). Internal validation was performed using 1,000 bootstrap resampling iterations, with an average absolute error of 0.036. Calibration curves demonstrated good agreement between the predicted and observed probabilities (Figure 3), with the calibration curve closely approximating the 45-degree reference line. DCA was performed to evaluate the clinical utility of the model (Figure 4), with higher net benefit indicating greater clinical usefulness. ROC curve analysis was performed to evaluate the predictive performance of the model (Figure 5), yielding an AUC of 0.783 (95% CI: 0.697–0.854).

To reduce the risk of overfitting, LASSO regression was performed to screen all 26 candidate variables included in the analysis. During this process, 10-fold cross-validation was used to optimize the model parameters and determine the key predictors. At lambda.1se, LASSO selected five variables with non-zero coefficients: lactate, postoperative opioid use, BMI, albumin, and age (Supplementary Figure S1). These variables were consistent with those retained in the multivariable logistic regression model. However, the LASSO coefficient paths became unstable when log(*λ*) < −5, with deviations initially decreasing and then increasing, suggesting a potential risk of overfitting if additional variables were included.

## Discussion

In this retrospective cohort study of patients with sepsis secondary to intra-abdominal infection, we identified several independent predictors of postoperative IAH, including advanced age, higher BMI, and elevated serum lactate levels, whereas postoperative oxycodone use and higher plasma albumin levels were associated with lower odds of postoperative IAH. However, this association does not imply causality due to the observational study design. Based on these predictors, a nomogram model was developed and demonstrated good discriminatory ability and calibration. These findings suggest that perioperative analgesic strategy may be associated with postoperative IAH risk and warrant further investigation.

IAH is increasingly recognised as a common and clinically important complication in critically ill patients, particularly in those with intra-abdominal infection or severe sepsis. Previous reviews have emphasized that increased intra-abdominal pressure can impair multiple organ systems by reducing venous return, compromising renal perfusion, and limiting diaphragmatic movement, ultimately contributing to respiratory and renal dysfunction (19–21). Clinical observations have shown that IAH frequently develops in critically ill patients receiving aggressive fluid resuscitation or experiencing intestinal edema and ileus (6, 7). Monitoring of intra-abdominal pressure has therefore been recommended as an important component of critical care assessment, particularly in patients with abdominal sepsis or postoperative complications (3, 5). In our study, patients who developed IAH also exhibited a higher incidence of acute kidney injury and required longer mechanical ventilation, findings consistent with previous clinical observations demonstrating that elevated intra-abdominal pressure contributes to organ dysfunction and adverse outcomes in ICU populations (4, 21). Physiologically, increased intra-abdominal pressure can elevate renal venous pressure and reduce renal perfusion, thereby impairing kidney function and promoting the development of acute kidney injury (22, 23). In addition, impaired diaphragmatic excursion and reduced lung compliance may aggravate respiratory failure and prolong mechanical ventilation in critically ill patients (24).

A notable finding of the present study is the observed association between postoperative oxycodone use and lower odds of postoperative IAH. Oxycodone is a semi-synthetic opioid that acts on both μ- and κ-opioid receptors and has been shown to provide effective analgesia for visceral pain. Several clinical studies have demonstrated that oxycodone has been reported to provide effective visceral analgesia in abdominal surgery settings and may attenuate postoperative stress responses and inflammatory marker levels (25, 26). Experimental research further suggests that oxycodone may exert anti-inflammatory effects by modulating immune responses and reducing inflammatory cytokine production (14, 27). These pharmacological characteristics may provide a possible biological explanation for the observed association; however, this hypothesis requires further investigation. Adequate analgesia may be associated with reduced abdominal wall tension and sympathetic activation, which could potentially influence intra-abdominal pressure regulation. Moreover, effective pain control may be associated with gastrointestinal functional changes that could influence intra-abdominal pressure. Similar observations have been reported in pediatric patients with appendicular peritonitis, in whom postoperative analgesic techniques influenced intestinal barrier function and intra-abdominal pressure (28). Furthermore, recent multicenter studies have demonstrated that oxycodone provides effective analgesia and a favorable safety profile in mechanically ventilated patients with respiratory failure, further supporting its potential role in critically ill populations (29). Collectively, these findings suggest that postoperative analgesic strategy may represent a potentially relevant factor associated with postoperative IAH risk. However, given the lack of a direct comparator group and the observational nature of this study, no conclusions regarding the superiority of oxycodone over other analgesic regimens can be drawn.

In addition to analgesic strategy, several physiological variables were identified as independent predictors of postoperative IAH in the present study. Elevated serum lactate was significantly associated with increased risk of IAH. Lactate is widely recognized as a marker of tissue hypoperfusion and metabolic stress in patients with sepsis and critical illness, and elevated lactate levels have consistently been associated with disease severity and poor prognosis (30). In septic patients, systemic inflammation and microcirculatory dysfunction can impair intestinal perfusion and compromise the integrity of the intestinal barrier, resulting in increased vascular permeability and interstitial edema (1, 2). These pathophysiological processes may contribute to intestinal swelling and fluid accumulation within the abdominal cavity, thereby increasing intra-abdominal pressure. Hypoalbuminaemia was also identified as an independent variable associated with postoperative IAH in our model. Albumin plays an important role in maintaining plasma oncotic pressure and limiting interstitial fluid accumulation. Reduced albumin levels may promote fluid leakage into the interstitial space and exacerbate visceral edema, which may in turn contribute to increased intra-abdominal pressure. Previous studies evaluating early fluid resuscitation strategies in sepsis have highlighted the physiological importance of albumin in maintaining vascular stability and limiting fluid extravasation (31).

Advanced age and increased BMI were also independently associated with postoperative IAH in the present study. Age-related physiological changes, including reduced abdominal wall compliance, impaired microcirculatory regulation, and increased prevalence of frailty, may predispose elderly patients to complications following major abdominal surgery (32). In addition, obesity is known to increase baseline intra-abdominal pressure due to increased visceral adiposity and reduced abdominal wall compliance. Previous observational studies have identified obesity as an important risk factor for intra-abdominal hypertension and abdominal compartment syndrome in critically ill patients (33). These findings are consistent with the results of our study and further support the inclusion of these variables in predictive models for postoperative IAH risk stratification. Collectively, our findings are broadly consistent with existing literature while extending current knowledge by demonstrating that postoperative analgesic strategy, including oxycodone use, may represent a potentially relevant factor associated with postoperative IAH risk.

This study has several limitations. First, as a single-center retrospective study, the results may be subject to selection bias and limited generalizability. Patient management strategies, perioperative care protocols, and analgesic practices may vary between institutions, which could influence the incidence and determinants of intra-abdominal hypertension. Therefore, future multicenter studies with larger sample sizes are required to externally validate the predictive model and confirm its generalizability. Second, some patients were transferred to other hospitals during treatment, which may have influenced the accuracy of postoperative hospital length of stay and in-hospital mortality outcomes. In addition, patients were not followed up after hospital discharge, preventing evaluation of long-term outcomes. Future studies should therefore include extended follow-up periods to investigate the long-term effects of analgesic strategies on chronic postoperative pain, functional recovery, and overall prognosis. Third, several potentially important intraoperative variables were not routinely recorded in the present study. These included pneumoperitoneum pressure and duration during laparoscopic surgery, intraoperative airway pressure during mechanical ventilation, intraoperative fluid administration, estimated blood loss, blood transfusion, cumulative fentanyl exposure and the number of rescue administrations. These factors may substantially influence abdominal wall compliance, bowel edema, fluid balance, and ultimately postoperative intra-abdominal pressure. Consequently, omission of these variables may have resulted in residual confounding and may have affected the predictive performance and generalizability of the nomogram. Consequently, omission of these variables may have resulted in residual confounding and may have affected the predictive performance and generalizability of the nomogram. Importantly, cumulative fentanyl exposure and the exact number and timing of rescue administrations were not consistently available in the retrospective records. Therefore, rescue fentanyl use was analyzed only as a binary variable, which may not have fully captured differences in total opioid exposure between patients and may have resulted in residual confounding. In addition, the observed association between oxycodone use and postoperative IAH may be affected by confounding by indication, as the choice of analgesic regimen was determined by clinicians rather than randomly assigned. Although relevant clinical variables were adjusted, residual confounding from unmeasured factors cannot be excluded. Future research should incorporate these variables to provide a more comprehensive understanding of the determinants of postoperative IAH. Finally, the relatively small number of outcome events (*n* = 49) compared with the initial number of candidate predictors (*n* = 26) raises a potential risk of model overfitting. Although LASSO regression with 10-fold cross-validation identified the same five predictors as the conventional multivariable model and 1,000 bootstrap resampling iterations were used for internal validation, these approaches cannot fully overcome the limitations imposed by the sample size. Therefore, the model should be considered exploratory and requires external validation in larger, independent cohorts before clinical application

## Conclusions

In conclusion, postoperative IAH is a clinically important complication in patients with sepsis secondary to intra-abdominal infection. This study identified advanced age, elevated BMI, and increased serum lactate as independent risk factors, whereas postoperative oxycodone analgesia and higher plasma albumin levels were associated with a reduced risk of IAH. A nomogram incorporating these variables demonstrated good predictive performance and may serve as a practical tool for early risk stratification in clinical practice. Early identification of high-risk patients may facilitate closer monitoring and risk stratification. Further prospective studies are required to determine whether specific analgesic strategies influence the development of postoperative IAH.

## Data Availability

The original contributions presented in the study are included in the article/Supplementary Material, further inquiries can be directed to the corresponding authors.

## Supplementary material

Supplementary Figure S1 The coefficient path plot and lambda curve of LASSO.
