# Supplementary material for: Development of a nomogram prediction model for postoperative intra-abdominal hypertension in patients with intra-abdominal infection sepsis based on analgesic drug selection
Source: Front Surg. 2026 Sep 11;13:1899876. doi: 10.3389/fsurg.2026.1899876 (PMC13613980; doi:10.3389/fsurg.2026.1899876)
Supplement: Supplementary file 1 [file Table1.docx]

Supplementary Table S1. Missing data patterns and handling strategies for variables included in the analysis

| Variable | Total n | Missing n | Missing % | Handling |
| --- | --- | --- | --- | --- |
| IAH | 116 | 0 | 0 | Not imputed |
| NT-proBNP, | 116 | 14 | 12.07 | Multiple imputation |
| HGB | 116 | 0 | 0 | Not imputed |
| ALB | 116 | 0 | 0 | Not imputed |
| T-Bil | 116 | 16 | 13.79 | Multiple imputation |
| ALT | 116 | 0 | 0 | Not imputed |
| AST | 116 | 0 | 0 | Not imputed |
| Cr | 116 | 7 | 6.03 | Multiple imputation |
| WBC | 116 | 0 | 0 | Not imputed |
| NEUT | 116 | 0 | 0 | Not imputed |
| LYMPH | 116 | 0 | 0 | Not imputed |
| PLT | 116 | 0 | 0 | Not imputed |
| CRP | 116 | 11 | 9.48 | Multiple imputation |
| Serum lactate | 116 | 5 | 4.31 | Multiple imputation |
